# Feasibility and Diagnostic Potential of Multimodal Neuro-Ophthalmic Biomarkers in Young-Onset Dementia and Functional Cognitive Disorder

**DOI:** 10.1016/j.xops.2026.101410

**Published:** 2026-09-17

**Authors:** Irem Karaer, Callum Hunt, Seema Teli, Reenette Savant, Vanessa Rodwell, Ha-Jun Yoon, Gail D.E. Maconachie, Zhanhan Tu, Qadeer Arshad, Elizabeta B. Mukaetova-Ladinska, Mervyn G. Thomas

**Affiliations:** 1 Ulverscroft Eye Unit, School of Psychology and Vision Sciences, University of Leicester, Leicester, United Kingdom; 2 School of Psychology and Vision Sciences, University of Leicester, Leicester, United Kingdom; 3 The Evington Centre, Leicester General Hospital, Leicester, United Kingdom; 4 Department of Ophthalmology, University Hospitals of Leicester NHS Trust, Infirmary Square, Leicester, United Kingdom

**Keywords:** Young onset dementia, Functional cognitive impairment, Culture-fair diagnosis, Neuro-ophthalmic biomarkers, Point-of-care assessment

## Abstract

**Purpose:**

To evaluate the feasibility and diagnostic utility of neuro-ophthalmic biomarkers as objective, culture-fair tools for distinguishing young-onset dementia (YOD) from functional cognitive disorder (FCD).

**Design:**

A cross-sectional observational study.

**Participants:**

Forty-five participants were recruited from a young-onset memory clinic, including cognitively normal controls (*n* = 18), individuals with FCD (*n* = 11), and patients with YOD (*n* = 16).

**Methods:**

Participants underwent point-of-care neuro-ophthalmic assessments, including virtual reality–based eye tracking (BulbiCAM) to measure saccadic and pupillary responses, and handheld electroretinography (ERG) (RETeval) to assess retinal ganglion cell function.

**Main Outcome Measures:**

Saccadic latency (horizontal and vertical), pupillometric parameters (constriction acceleration and velocity, relative afferent pupillary defect, and baseline diameter), and ERG photopic negative response (PhNR) amplitude and latency.

**Results:**

Saccadic latency was significantly prolonged in YOD compared with nondementia participants for horizontal (*P* = 0.0036) and vertical (*P* = 0.033) tasks, with 3-group analysis showing a stepwise increase from controls to FCD to YOD. Dynamic pupillometry also differentiated YOD, with reduced constriction acceleration (*P* = 0.0003) and velocity (*P* = 0.02), and similarly showed a stepwise decline from controls through FCD to YOD. Constriction acceleration showed the strongest single-marker performance (area under the curve [AUC] = 0.83), followed by horizontal saccadic latency (AUC = 0.77). Combining these 2 measures improved discrimination (AUC = 0.84), with high specificity (92.6%). Electroretinography PhNR measures showed nonsignificant trends toward reduced amplitude and delayed latency in cognitively impaired groups. Participant feedback supported feasibility, with over 93% reporting comfort and acceptability.

**Conclusions:**

Noninvasive neuro-ophthalmic assessments are feasible in memory clinic settings and show promise as rapid, objective, and culture-fair biomarkers for early detection of YOD. Dynamic pupillometry and saccadic latency, in particular, demonstrate potential for differentiating YOD from nondementia conditions, while their combined model showed high specificity, supporting possible value as a rule-in adjunct in clinical assessment. Larger, longitudinal studies are required to validate diagnostic thresholds and clinical implementation.

**Financial Disclosure(s):**

The author has no/the authors have no proprietary or commercial interest in any materials discussed in this article.

Dementia is a growing global health challenge, with nearly 10 million new cases reported each year and major personal, societal, and economic consequences.^1^^,^^2^ This is expected to more than double by 2040, further threatening independence and quality of life.^2^ Early and accurate diagnosis is essential to enable timely treatment, care planning, and psychosocial support, which may slow functional decline and reduce caregiver strain.^3^ Although dementia is commonly associated with older age, young-onset dementia (YOD), defined as dementia before the age of 65, is increasingly recognized and presents distinct challenges for employment, family life, and social functioning.^4^^,^^5^

One key diagnostic challenge for dementia lies in the differential diagnosis between YOD and functional cognitive disorder (FCD). Individuals with FCD present with persistent but internally inconsistent cognitive symptoms not attributable to any identifiable brain damage or disease and may arise from comorbid conditions.^6^ Misclassification of FCD as YOD may lead to unnecessary referrals and investigations, inappropriate treatment, and significant social and psychological consequences of diagnostic uncertainty.^7^ However, current diagnostic methods remain limited: cognitive tests are influenced by language and education, while neuroimaging and biomarker assays are invasive, costly, and not widely accessible. As a result, many patients, especially from minority or lower-education backgrounds, experience delayed or missed diagnoses, highlighting the need for reliable and equitable biomarkers.^8^, ^9^, ^10^

Visual-system biomarkers, including oculomotor and pupillary responses, show strong potential for dementia diagnosis as rapid, noninvasive indicators of neurodegenerative change.^11^^,^^12^ Prolonged saccadic latency reflects deficits in attentional and executive networks, which are affected early in dementia and are also markers of attentional deficits, a feature highly relevant in patients with FCD.^8^^,^^13^ Altered pupillary light reflex dynamics, particularly constriction velocity and acceleration, reflect cholinergic dysfunction within the Edinger–Westphal nucleus, a region known to degenerate in Alzheimer disease (AD) and related dementias, providing sensitive indicators of autonomic and CNS decline.^14^^,^^15^ In addition, retinal electrophysiology, particularly the photopic negative response (PhNR) of the electroretinography (ERG), offers another promising noninvasive biomarker, providing direct assessment of retinal ganglion cell function, often impaired in neurodegenerative diseases including AD.^16^^,^^17^

These neuro-ophthalmic measures are objective, noninvasive, and language-independent, supporting rapid and scalable approaches to early and equitable dementia diagnosis and monitoring at the point of care.^18^ Despite their promise, neuro-ophthalmic biomarkers remain underused due to technical, validation, and implementation challenges.^19^ Moreover, while these biomarkers have been studied individually, little is known about their combined feasibility and acceptability in routine clinical settings.

To evaluate the feasibility, tolerability, and diagnostic potential of a multimodal, noninvasive neuro-ophthalmic assessment battery (including saccadic latency, pupillometry, and handheld ERG), we integrated these point-of-care tests into routine memory clinic (<65 years). We then assessed whether this brief, culture-fair, and language-independent biomarker panel could distinguish YOD from nondementia groups (controls and FCD). Additionally, we examined whether these biomarkers differed significantly across diagnostic categories and explored their potential to differentiate FCD from early neurodegenerative disease.

## Methods

### Participants

A total of 45 age-matched participants were recruited from the young-onset memory clinic at the Leicester General Hospital, comprising controls (*n* = 18), FCD (*n* = 11), and YOD (*n* = 16). Across the 3 study groups, participants were of comparable age, with mean values of 60.7 ± 6.9 years in the control group, 60.2 ± 5.5 years in the FCD group, and 61.4 ± 2.9 years in the YOD group. Overall, the combined cohort (n = 45) had a mean age of 60.8 ± 5.4 years. The sex distribution was also broadly similar, with females representing 56% of the control group, 55% of the FCD group, and 38% of the YOD group, resulting in an overall female proportion of 49% across all participants. Clinical diagnoses of FCD and YOD were established based on cognitive assessment, neuroimaging, and blood investigations and confirmed by a consultant psychiatrist. Patient demographics, diagnostic profiles, neuroimaging findings, cognitive scores, and educational backgrounds are summarized in Table 1. Anticholinergic burden was quantified for patient participants using the Anticholinergic Cognitive Burden scale, based on the medications recorded in their clinical notes at the time of assessment. Individual drug scores were summed to produce a total Anticholinergic Cognitive Burden score, and a total score of ≥ 3 was taken to indicate clinically meaningful anticholinergic exposure.

**Table 1:** Demographic and Clinical Characteristics of YOD, FCD, and Control Participants

Participant	Age	Sex	Ethnicity	Diagnosis	Neuroradiology	ACE-III	RUDAS	ACB	Education
YOD									
D1	64	F	White-British	Alzheimer disease	MRI	61/100	20/30	0	GCSE
D2	66	M	Persian	Mixed dementia (AD and VaD)	MRI	59/100	NA	0	GCSE
D3	56	F	White-British	Alzheimer disease	MRI/FDG-PET	61/100	NA	1	GCSE
D4	64	F	White-British	Alzheimer disease	MRI	34/100	2/30	0	GCSE
D5	64	M	White-British	Vascular dementia	MRI	75/100	20/30	2	University
D6	63	M	White-British	Alzheimer disease	MRI	NA	14/30	0	University
D7	60	M	Asian-Indian	Parkinson disease dementia autism spectrum disorder	MRI	NA	21/30	0	Few years
D8	61	M	White-British	Parkinson disease dementia	MRI	NA	16/30	3	GCSE
D9	65	F	White-British	Alzheimer disease	FDG-PET	NA	17/30	1	University
D10∗	58	M	Mongolian	Poststroke dementia	MRI	NA	NA	1	Apprenticeship
D11	62	M	Asian-Indian	Alzheimer disease	FDG-PET	63/100	NA	1	University
D12	61	M	White-British	Dementia with Lewy body	MRI/FDG-PET/DAT	91/100	NA	1	GCSE
D13	57	F	White-British	Alzheimer disease	MRI/FDG-PET	72/100	21/30	0	University
D14	60	M	White-British	Alzheimer disease	FDG-PET	73/100	NA	0	GCSE
D15	59	M	White-British	Alzheimer disease	FDG-PET	59/100	24/30	0	University
D16	63	F	White-British	Alzheimer disease	MRI	NA	13/30	1	GCSE
FCD									
FCD1	62	M	White-British	Andropause	MRI/FDG-PET	76/100	24/30	1	GCSE
FCD2	62	M	Asian-Indian	Lack of sleep	MRI	NA	22/30	4	1 year of schooling
FCD3	62	M	White-British	Unregulated diabetes	MRI/FDG-PET	68/100	21/30	0	GCSE
FCD4	46	F	White-British	Memory problems secondary to bile acid malabsorption or diarrhea	MRI	90/100	28/30	3	University
FCD5	63	F	White-British	Memory problems secondary to anticholinergic drugs (ACB>5)	MRI	87/100	28/30	5	GCSE
FCD6	56	M	White-British	Autism spectrum disorder (no dementia)	MRI	99/100	NA	3	GCSE
FCD7	65	F	White-British	Fibromyalgia	FDG-PET	79/100	NA	2	University
FCD8	57	F	Asian-Pakistani	Depression	MRI	NA	21/30	3	Few years
FCD9	64	F	British Asian-Filipino	Postchemo/hypothyroidism	MRI	81/100	26/30	0	16 years (age at leaving school)
FCD10	65	F	Asian-Pakistani	Depression	MRI/FDG-PET	NA	20/30	1	No education
FCD11	60	M	Mixed-Black British	Personality traits/depression	MRI/FDG-PET	76	22/30	1	GCSE
Control									
CO1	52	F	White-British	No dementia (CDR: 0/3)	NA	NA	NA	NA	NA
CO2	58	M	Asian-Indian	No dementia (CDR: 0/3)	NA	NA	NA	NA	NA
CO3	66	M	White-Other	No dementia (CDR: 0/3)	NA	NA	NA	NA	NA
CO4	52	F	White-British	No dementia (CDR: 0/3)	NA	NA	NA	NA	NA
CO5	70	M	White-British	No dementia (CDR: 0/3)	NA	NA	NA	NA	NA
CO6	55	M	Asian-Indian	No dementia (CDR: 0/3)	NA	NA	NA	NA	NA
CO7	59	F	White-British	No dementia (CDR: 0/3)	NA	NA	NA	NA	NA
CO8	66	M	White-British	No dementia (CDR: 0/3)	NA	NA	NA	NA	NA
CO9	55	F	Asian-Indian	No dementia (CDR: 0/3)	NA	NA	NA	NA	NA
CO10	78	F	White-British	No dementia (CDR: 0/3)	NA	NA	NA	NA	NA
CO11	56	M	White-British	No dementia (CDR: 0/3)	NA	NA	NA	NA	NA
CO12	61	F	White-British	No dementia (CDR: 0/3)	NA	NA	NA	NA	NA
CO13	55	F	White-British	No dementia (CDR: 0/3)	NA	NA	NA	NA	NA
CO14	61	M	White-British	No dementia (CDR: 0/3)	NA	NA	NA	NA	NA
CO15	68	M	White-British	No dementia (CDR: 0/3)	NA	NA	NA	NA	NA
CO16	57	F	Mixed-Black British	No dementia (CDR: 0/3)	NA	NA	NA	NA	NA
CO17	58	F	White-British	No dementia (CDR: 0/3)	NA	NA	NA	NA	NA
CO18	65	F	White-other	No dementia (CDR: 0/3)	NA	NA	NA	NA	NA

ACB = Anticholinergic Cognitive Burden Score (higher scores indicate greater cumulative anticholinergic exposure); ACE-III = Addenbrooke’s Cognitive Examination III; AD = Alzheimer disease; CDR = Clinical Dementia Rating; DAT scan = dopamine transporter scan; FCD = functional cognitive disorder; FDG-PET = fluorodeoxyglucose positron emission tomography; MRI = magnetic resonance imaging; NA = not applicable; RUDAS = Rowland Universal Dementia Assessment Scale; VaD = vascular dementia; YOD = young-onset dementia.

The table presents individual-level data including age, sex, ethnicity, primary diagnosis, neuroimaging modality, cognitive assessment scores, anticholinergic burden, and educational background. Clinical features and inclusion criteria for controls are explained below.

∗ D10 had poststroke motor aphasia and could not be assessed with the routine cognitive tests used in the memory clinic, Clinical Dementia Rating score corresponding to 2/3.

Controls (*n* = 18) were recruited through convenience sampling from age-matched spouses or relatives of memory clinic patients, as well as clinical staff volunteers. Control participants had no subjective cognitive complaints or history of neurological or psychiatric disorders and demonstrated normal performance on cognitive screening. None of them showed decline from their previous usual cognitive level, and they achieved a Clinical Dementia Rating score of 0/3, confirming absence of cognitive impairment. Participant demographics are explained in Table 1.

All participants underwent brief ophthalmological screening. The exclusion criteria included advanced cataracts, glaucoma, or significant retinal pathology that could confound results. Refractive errors were common but were corrected through diopter adjustment on the eye-tracking device to ensure optimal tracking performance and data quality.

The study was conducted in accordance with the principles of the Declaration of Helsinki. Ethical approval for this study was obtained from the East Midlands – Nottingham 2 Research Ethics Committee (REC reference: 21/EM/0017, protocol version: 0772) and the UK Health Research Authority (IRAS ID: 279309). All participants provided informed consent prior to inclusion.

### Study Design and Data Collection

Participants completed a single study visit during their routine memory clinic appointments (10–15 min), and which comprised 3 noninvasive neuro-ophthalmic tests designed to evaluate complementary aspects of visual and neural function.

Saccadic eye movements and pupillometry were performed using a virtual reality–based eye-tracking system BulbiCAM (BulbiTech AS), capable of high-frequency recording of dynamic pupillary responses. BulbiCAM’s technical reliability and intervisit reproducibility for pupillometry, and intervisit reproducibility for saccadic metrics, have been established previously in a separate validation study in healthy participants.^20^ BulbiCAM^21^ is a Conformité Européenne/European Conformity–marked and Food and Drug Administration–registered eye-tracking system that employs artificial intelligence-integrated software to analyze image data captured by its cameras.

Retinal electrophysiology was assessed using a handheld ERG device RETeval (LKC Technologies), a portable, handheld ERG device compliant with International Society for Clinical Electrophysiology of Vision standards. It utilizes skin electrodes to obtain PhNR recordings without the need for pharmacological pupil dilation.^22^

This combined multimodal protocol was selected to provide objective, reproducible, and time-efficient measures of oculomotor, pupillary, and retinal function in a clinical population. Figure 1 illustrates the experimental set-up, including the virtual reality eye tracker test station, real-time test screen, and ERG device.

**Figure 1:**
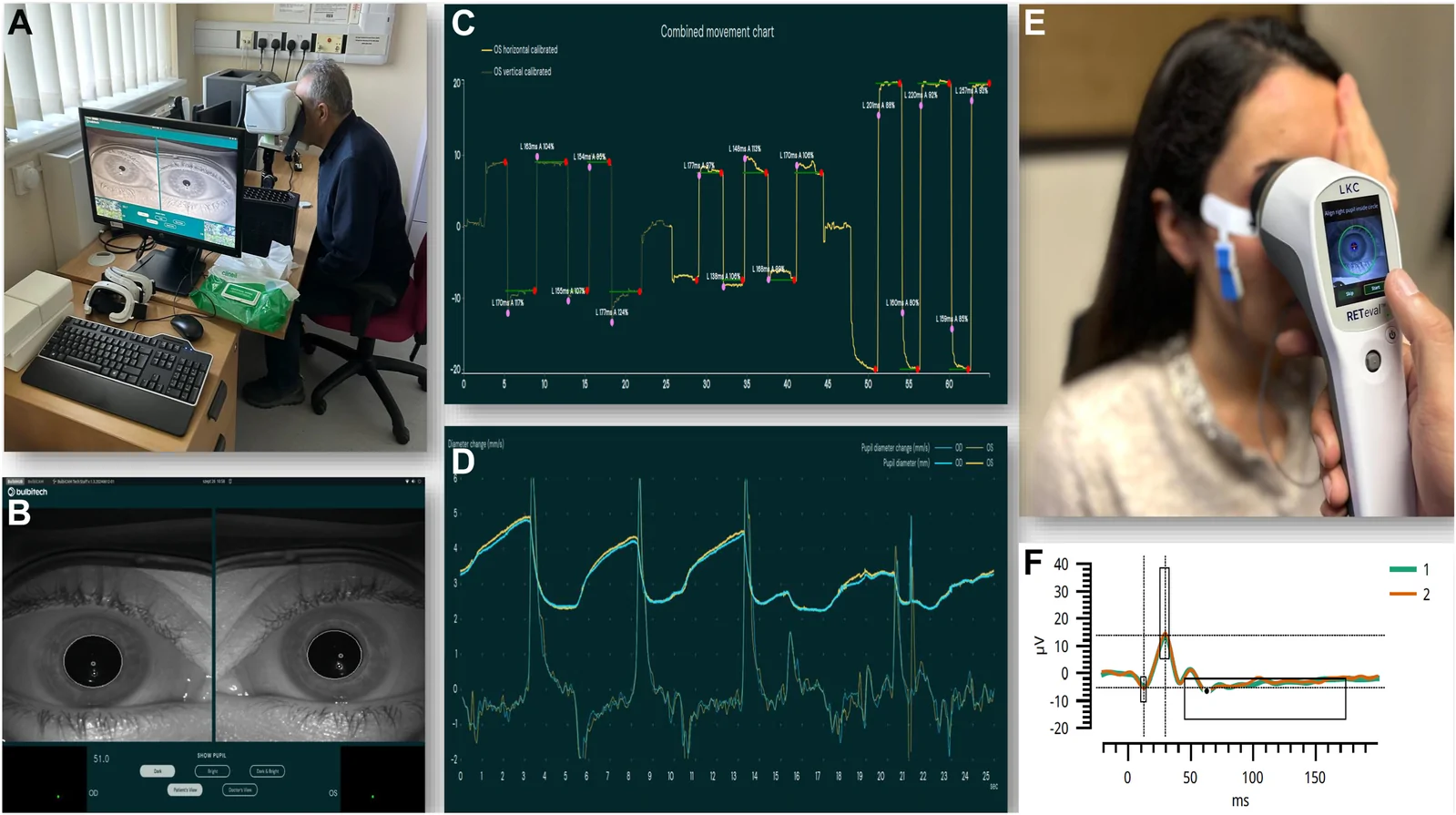
Test set-up at the dementia clinic and representative test outputs. **(A)** Virtual reality–based eye-tracking device setup with a participant undergoing assessment. **(B)** Real-time test screen from the eye tracker. **(C)** Example of saccade test output. **(D)** Example of pupillometry output graphs. **(E)** Handheld RETeval ERG device. **(F)** Representative ERG-PhNR output trace. ERG = electroretinography; PhNR = photopic negative response.

### Participant Experience

Participants completed a postassessment questionnaire evaluating comfort, duration, brightness, visual disturbance, and fatigue using a 5-point Likert scale (Supplementary Data 1, available at www.ophthalmologyscience.org).

### Data Set and Statistical Analysis

Saccade and pupillometry test protocols and eye-tracking calibration process are enclosed in Supplementary Data 2, available at www.ophthalmologyscience.org. For pupillometry, data from 9 of the 11 participants with FCD were included in the final analysis, with 2 recordings excluded during the original data-cleaning process because they did not meet data-quality requirements. Retinal electrophysiology was assessed by extracting PhNR amplitudes and latencies for analysis. Figure 1F illustrates representative waveforms from the PhNR test.

Data normality was assessed using the Shapiro–Wilk test. Between-group comparisons were performed using unpaired *t* tests or Mann–Whitney *U* tests, with 3-group comparisons assessed using Kruskal–Wallis tests with Dunn post hoc analysis. For acceleration data, 1-way analysis of variance (ANOVA) with Tukey post hoc testing was applied after verifying homogeneity of variances using Bartlett and Brown–Forsythe tests.

Receiver operating characteristic (ROC) curve analyses were performed to evaluate diagnostic performance in distinguishing YOD from nondementia participants. The area under the curve (AUC), sensitivity, specificity, and optimal cutoffs (Youden Index) were calculated.

To assess the combined diagnostic potential of the biomarkers, a multiple regression model including pupillary constriction, acceleration, and horizontal saccadic latency was constructed, and the predicted probabilities were used to generate a combined ROC curve. The AUC, sensitivity, specificity, and classification accuracy were calculated to evaluate the performance of the combined model.

A priori sample size estimation was based on the primary outcome measure, horizontal saccadic latency. Using pilot data, we anticipated a moderate-to-large between-group effect (Cohen’s *d* ≈ 0.65) for the comparison between YOD and nondementia participants (controls + FCD), with α = 0.05 and power = 0.80. This indicated a target of approximately 15 participants per group. Our final sample (controls + FCD: *n* = 29; YOD: *n* = 14) was therefore considered sufficient to detect clinically meaningful differences in saccadic latency.

Given the central origin of the neurodegenerative and functional conditions investigated, which result in bilateral and largely symmetrical ocular manifestations,^15^ data from the left eye were analyzed. To confirm that single-eye analysis was appropriate for the central, bilaterally symmetric conditions studied here, intereye agreement for the primary pupillary parameters was assessed on paired right- and left-eye data using the intraclass correlation coefficient (2-way random-effects, absolute agreement, and single measures). Agreement was moderate-to-good for both constriction velocity (intraclass correlation coefficient = 0.76, 95% confidence interval [CI] 0.59–0.86) and constriction acceleration (intraclass correlation coefficient = 0.73, 95% CI 0.54–0.85; both *P* < 0.001).

Statistical significance was set at *P* < 0.05. Statistical analysis was performed using GraphPad Prism 10.2.3.

## Results

### Primary Outcome: Saccadic Eye Movements

#### Two-Group Comparison (YOD *vs*. Nondementia)

Saccadic latency was significantly prolonged in YOD compared with the combined nondementia (controls + FCD) group for both horizontal (20°) and vertical (9°) paradigms. Unpaired *t* tests revealed significant delays in horizontal saccades (*P* = 0.0036) and vertical saccades (*P* = 0.033) in the YOD group (Fig. 2A, B).

**Figure 2:**
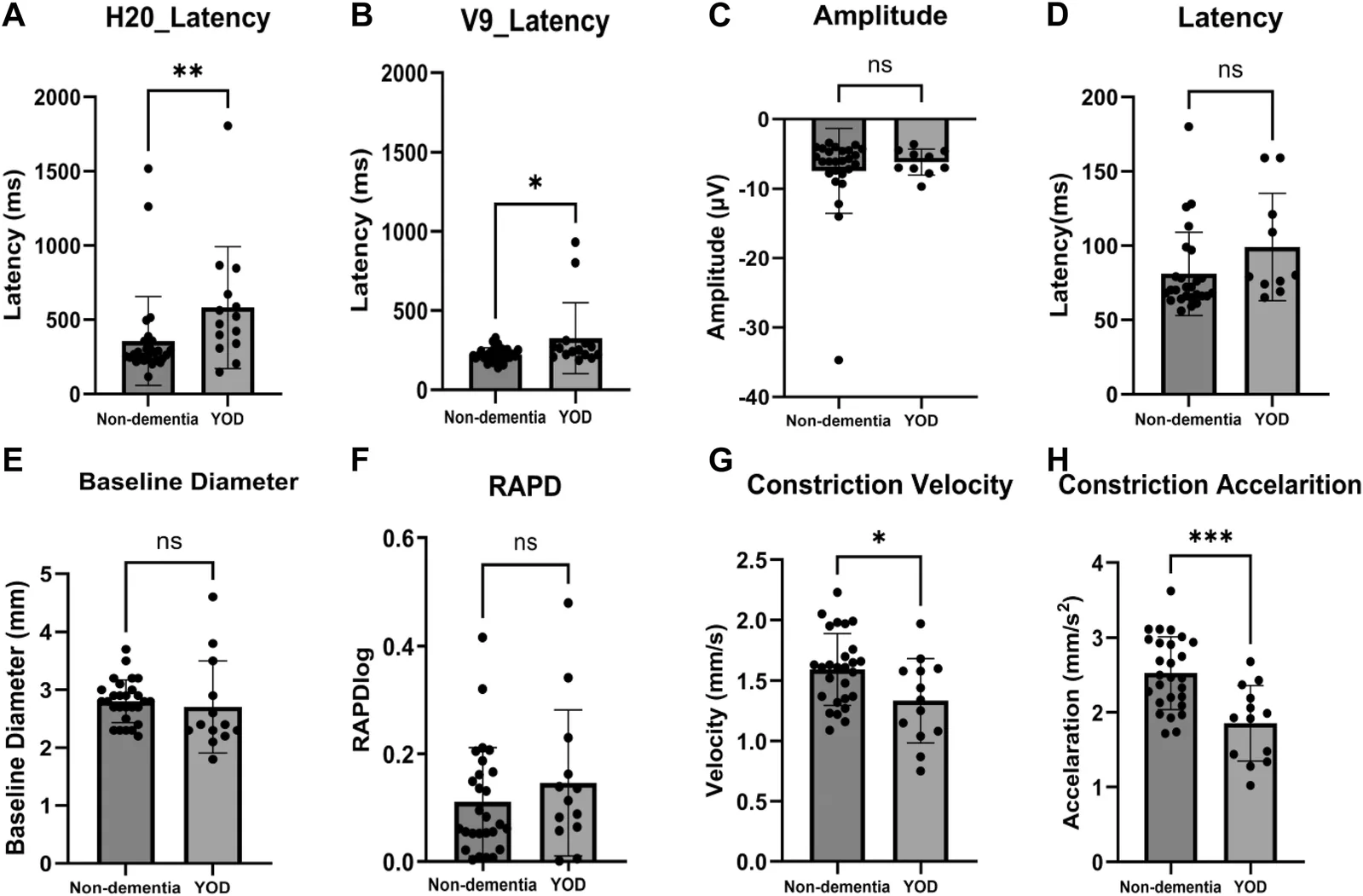
Group-wise comparisons of neuro-ophthalmic biomarkers across diagnostic groups. Each subplot **(A–H)** illustrates the distribution of a specific biomarker across participants without dementia, versus those with YOD. Bars represent mean ± standard error, and individual data points are shown as dots. Statistical comparisons were performed using unpaired *t* tests or Mann–Whitney *U* tests for 2-group comparisons (nondementia vs. YOD). **(A–B)** Horizontal (20°) and vertical (9°) saccadic latency show delayed responses in the YOD group compared with nondementia group. **(C–D)** Photopic negative response amplitude and latency show no significant differences, although values tend to be lower in YOD relative to those without dementia. **(E–H)** Pupillometry metrics: no significant differences were observed in baseline diameter or RAPD, whereas constriction velocity and acceleration were significantly reduced in YOD. Asterisks indicate levels of significance (∗P < 0.05, ∗∗P < 0.01, ∗∗∗P < 0.001). ns = not significant; RAPD = relative afferent pupillary defect; YOD = young-onset dementia.

#### Three-Group Analysis (Controls, FCD, and YOD)

Multigroup comparison revealed significant differences in both vertical (Kruskal–Wallis: χ^2^ = 8.06, df = 2, *P* = 0.018) and horizontal (χ^2^ = 8.79, df = 2, *P* = 0.012) saccadic latency. Post hoc Dunn tests demonstrated that YOD participants exhibited significantly prolonged latencies compared with controls in both vertical (*P* = 0.020) and horizontal (*P* = 0.010) tasks. Importantly, the mean latency values demonstrated a progressive increase from controls through FCD to YOD (Fig. 3), with FCD showing intermediate values that did not differ significantly from either controls (vertical: *P* = 0.176; horizontal: *P* = 1.00) or YOD (vertical: *P* = 1.00; horizontal: *P* = 0.205). This gradient pattern suggests that saccadic latency may capture a continuum of functional decline across cognitive states.

**Figure 3:**
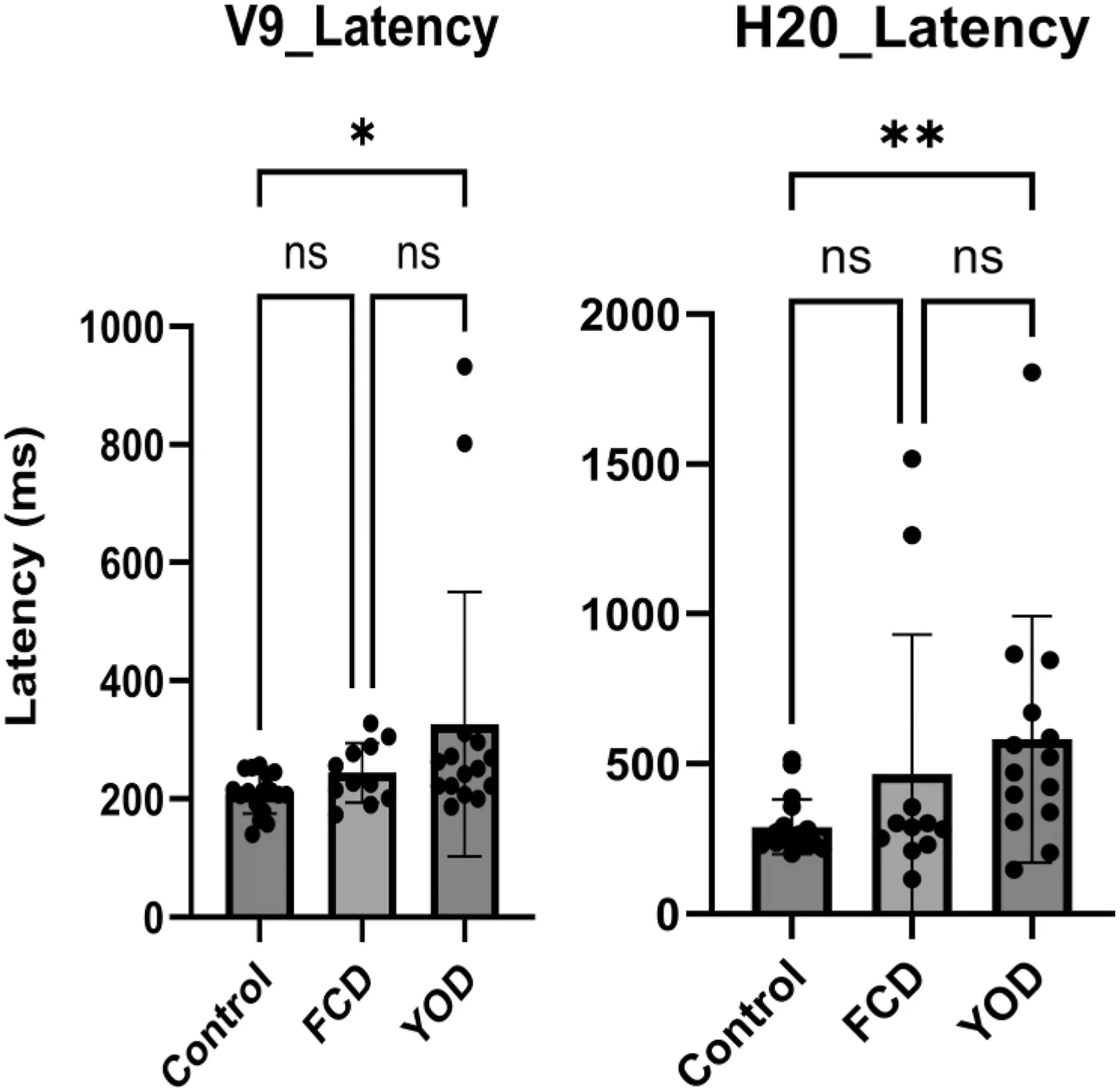
Saccadic eye movement latency across groups. Latency of saccadic eye movements in vertical (V9_Latency) and horizontal (H20_Latency) tasks across the control, FCD, and YOD groups. Data are presented as mean ± SEM with individual data points overlaid. Kruskal–Wallis tests revealed significant group differences for both vertical (χ^2^ = 8.06, df = 2, *P* = 0.018) and horizontal (χ^2^ = 8.79, df = 2, *P* = 0.012) tasks. Post hoc Dunn tests showed that YOD participants exhibited significantly prolonged latencies compared with controls in both tasks (*P* = 0.020 and *P* = 0.010, respectively). No significant differences were observed between the FCD and control groups or between the FCD and YOD groups. Asterisks indicate levels of significance (∗*P* < 0.05, ∗∗*P* < 0.01). FCD = functional cognitive disorder; ns = not significant; SEM = standard error of the mean; YOD = young-onset dementia.

### Secondary Outcome: Pupillometry

#### Two-Group Comparison (YOD *vs*. Nondementia)

The dynamic pupillary parameters demonstrated significant group differences, while the static measures showed nonsignificant trends. Baseline pupil diameter and relative afferent pupillary defect (RAPD) did not differ significantly between YOD and nondementia groups (*P* = 0.13 and *P* = 0.42, respectively), although trends toward smaller baseline diameters and higher RAPD values were observed in YOD (Fig. 2E, F). In contrast, constriction dynamics were markedly impaired in YOD. Constriction velocity was significantly reduced (*P* = 0.02), and constriction acceleration was markedly lower (*P* = 0.0003) in YOD compared with nondementia (Fig. 2G, H), indicating parasympathetic dysfunction.

#### Three-Group Analysis (Controls, FCD, and YOD)

Multigroup analysis reinforced the discriminatory value of dynamic pupillary measures. Neither baseline diameter (Kruskal–Wallis: χ^2^ = 2.96, *P* = 0.228) nor RAPD (χ^2^ = 0.81, *P* = 0.667) differed significantly between groups, though both showed stepwise decline from controls to FCD to YOD (Fig. 4A, B). The mean constriction velocity differed significantly across groups (Kruskal–Wallis: χ^2^ = 8.13, *P* = 0.017), with post hoc analysis showing YOD participants had significantly slower velocity than controls (*P* = 0.018). A clear stepwise decline was evident from controls through FCD to YOD (Fig 4C). The mean constriction acceleration demonstrated even more robust group differences (1-way ANOVA: F(2,37) = 8.83, *P* = 0.0007, R^2^ = 0.32). Tukey post hoc analysis revealed markedly reduced acceleration in YOD compared with controls (*P* = 0.0005), while the FCD–YOD comparison approached significance (*P* = 0.051) (Fig. 4D).

**Figure 4:**
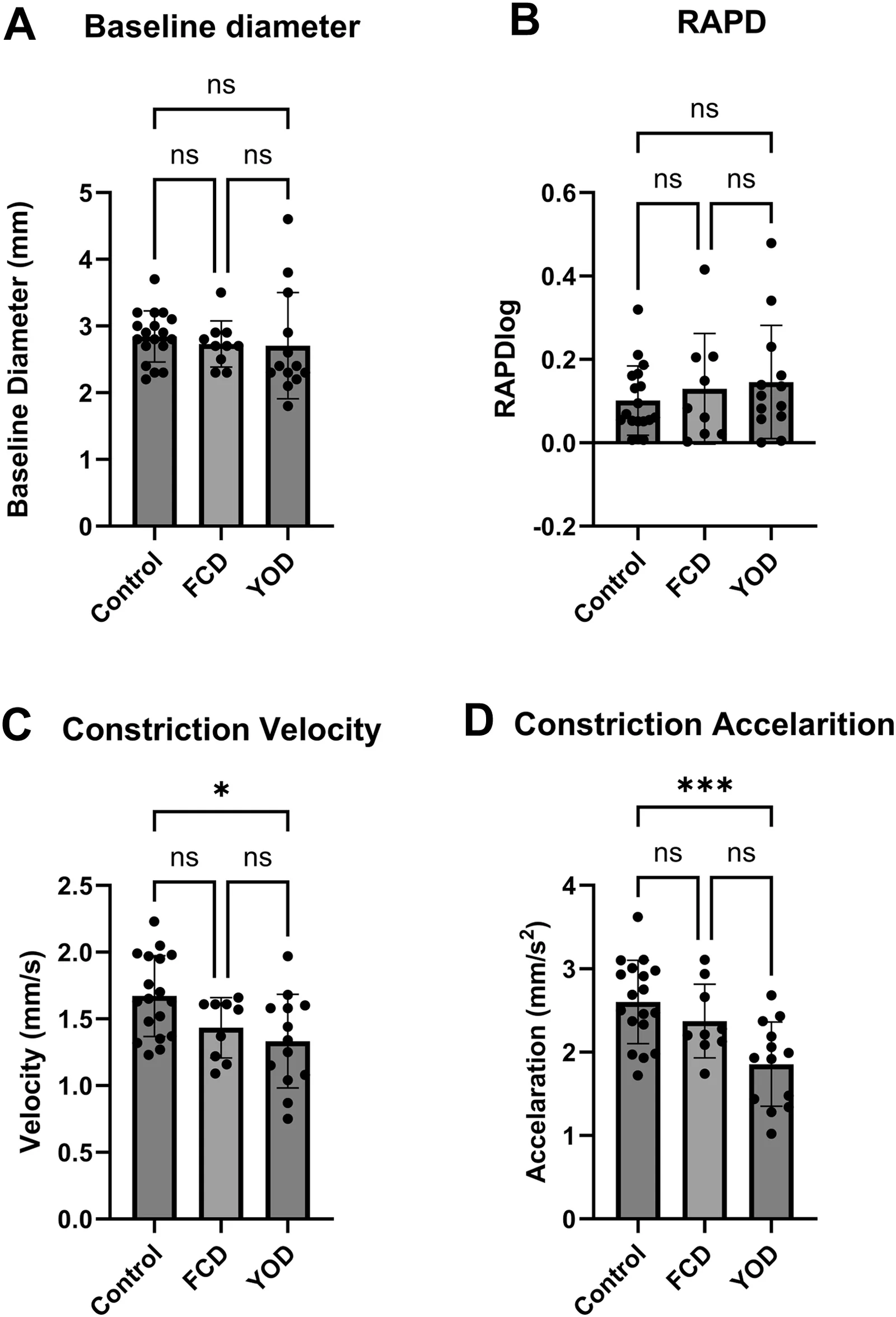
Pupillometric parameters across cognitive groups. **(A)** Baseline pupil diameter (mm) showing nonsignificant differences (ns) between control, FCD, and YOD groups. **(B)** Relative afferent pupillary defect (log scale) values also did not differ significantly across groups. **(C)** Mean constriction velocity (mm/s) demonstrated a significant reduction in the YOD group compared to controls (∗*P* < 0.05), with no significant difference between FCD and the other groups. **(D)** Mean constriction acceleration (mm/s^2^) was significantly lower in YOD compared to controls (∗∗∗*P* < 0.001), with FCD showing intermediate values not significantly different from either group. Bar plots represent group means ± SEM. Individual data points are shown as black dots. Statistical significance was assessed using Kruskal–Wallis and analysis of variance tests with post hoc comparisons. FCD = functional cognitive disorder; ns = not significant; RAPD = relative afferent pupillary defect; SEM = standard error of the mean; YOD = young-onset dementia.

### Diagnostic Performance

Receiver operating characteristic analyses showed moderate discriminative ability across parameters for distinguishing dementia from nondementia participants. Pupillary constriction acceleration showed the highest diagnostic performance (AUC = 0.83), followed by horizontal saccadic latency (AUC = 0.77). Pupillary constriction velocity (AUC = 0.72) and vertical saccadic latency (AUC = 0.70) demonstrated lower specificity despite relatively high sensitivity.

Sensitivity, specificity, optimal cutoff values (Supplementary Table S3), and ROC curves (Supplementary Figure S3) are provided in Supplementary Data 3, available at www.ophthalmologyscience.org.

### Combined Biomarker Analysis

A combined biomarker model for pupillary constriction acceleration and horizontal saccadic latency demonstrated good discriminatory performance (AUC = 0.84, 95% CI: 0.70–0.98, *P* = 0.0006). Classification analysis showed an overall accuracy of 82.5%, with high specificity (92.6%) and moderate sensitivity (61.5%). Detailed results (Supplementary Table S4 and Supplementary Figure S4) are presented in Supplementary Data 4, available at www.ophthalmologyscience.org.

### Exploratory Outcome: ERG-PhNR

#### Two-Group Comparison (YOD *vs*. Nondementia)

Electroretinography photopic negative response analysis revealed nonsignificant trends consistent with inner retinal dysfunction in YOD. Neither amplitude nor latency differed significantly between YOD and nondementia groups (amplitude: *P* = 0.95; latency: *P* = 0.054). However, both parameters showed directionally consistent trends toward reduced amplitude and delayed latency in YOD relative to nondementia (Fig. 2C, D).

#### Three-Group Analysis (Controls, FCD, and YOD)

Multigroup comparison did not reveal statistically significant differences in PhNR amplitude (Kruskal–Wallis: χ^2^ = 3.15, *P* = 0.207) or latency (χ^2^ = 4.17, *P* = 0.124), with post hoc tests confirming no significant pairwise differences (all *P* > 0.12). Nevertheless, a visual trend toward increasing latency from controls through FCD to YOD was apparent (Fig. 5), consistent with the pattern observed in other biomarkers. Post hoc power analysis indicated that approximately 45 participants per group would be required to detect significant differences in PhNR parameters, suggesting the current study was underpowered for this exploratory outcome.

**Figure 5:**
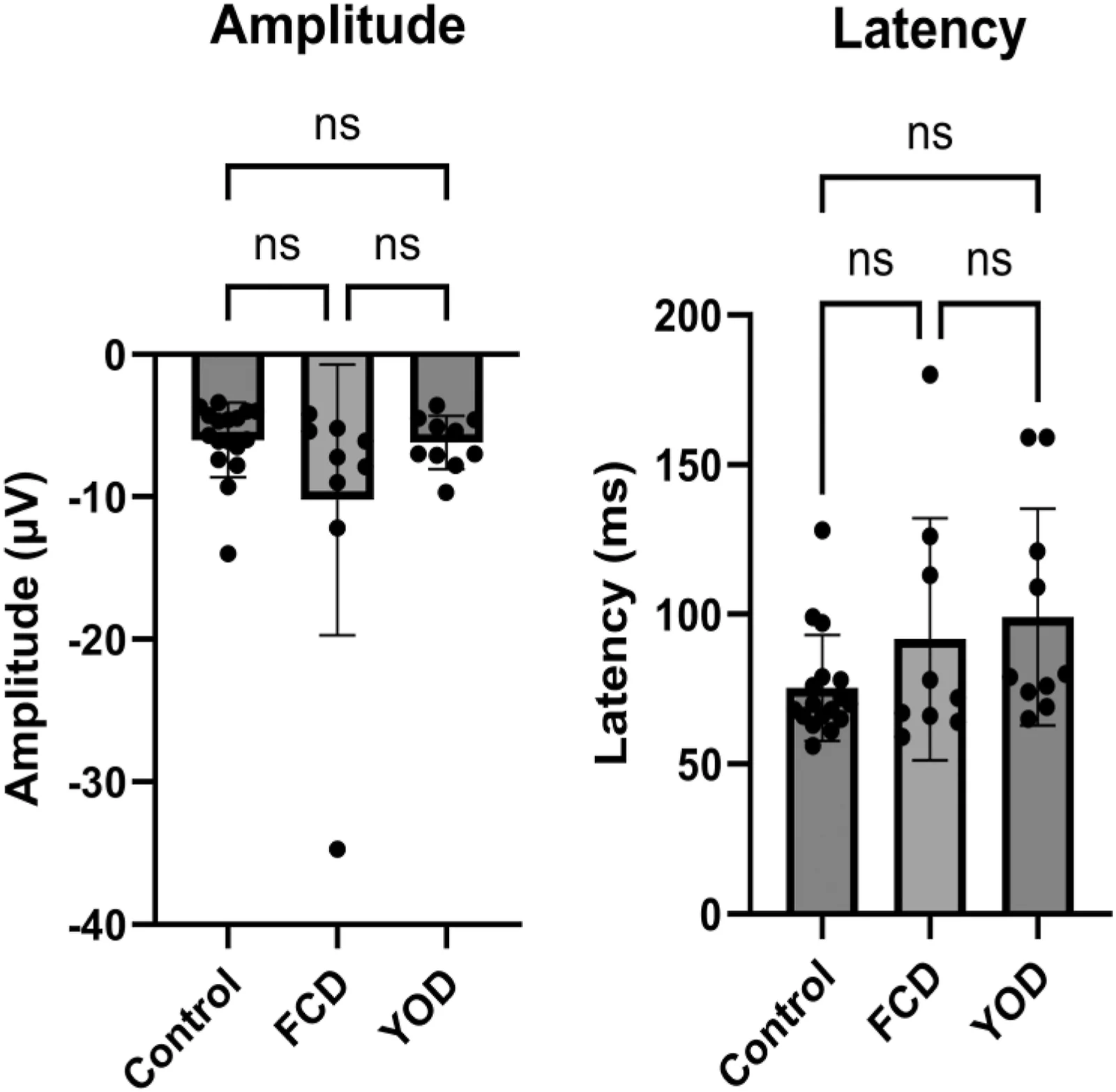
Electroretinography photopic negative response amplitude and latency across groups. Amplitude (left) and latency (right) of the ERG-PhNR response in control, FCD, and YOD groups. Data are shown as mean ± SEM with individual data points overlaid. Kruskal–Wallis tests showed no statistically significant differences in amplitude (χ^2^ = 3.15, df = 2, *P* = 0.207) or latency (χ^2^ = 4.17, df = 2, *P* = 0.124). ERG-PhNR = electroretinography photopic negative response; FCD = functional cognitive disorder; ns = not significant; SEM = standard error of the mean; YOD = young-onset dementia.

### Participant Experience and Feasibility

The postassessment acceptability questionnaire was completed by 30 of the 45 participants (67%). Completion rates were 83% in controls, 55% in the FCD group, and 56% in the YOD group and did not differ significantly across groups (χ^2^(2) = 3.76, *P* = 0.15). Among those who completed it, acceptability was consistently high: more than 90% rated the examination as comfortable, appropriately timed, and of tolerable brightness, with responses comparable across groups (Supplementary Table S1). Full Likert distributions are provided in Supplementary Figure S1 (Supplementary Data 1).

## Discussion

We demonstrate the feasibility and diagnostic potential of multimodal, noninvasive neuro-ophthalmic assessments in a routine memory clinic. Saccadic and pupillometric measures emerged as the most sensitive markers of early neurodegenerative change, successfully differentiating YOD from FCD and controls. Specifically, prolonged horizontal saccadic latency in YOD group reflected impaired frontoparietal network efficiency, while reduced pupillary constriction velocity and acceleration indicated early parasympathetic dysfunction consistent with cholinergic deficit. Both measures differentiated YOD from FCD and controls, suggesting potential for biomarker development. Importantly, ROC analyses demonstrated moderate discriminatory performance across individual biomarkers, with pupillary constriction acceleration showing the highest accuracy. Combining autonomic and cortical markers resulted in only a modest improvement in overall performance, with pupillary constriction acceleration driving most of the discrimination. However, the combined model showed higher specificity, suggesting improved identification of nondementia cases. This highlights the potential of integrating complementary systems to enhance early detection of neurodegenerative disease. Additionally, the trend toward prolonged ERG-PhNR latency in YOD supports subtle inner retinal involvement, consistent with PhNR changes seen in neurodegeneration, although confirmation in larger cohorts is needed. Furthermore, high participant acceptability supports their suitability for integration into routine diagnostic pathways.

Prolonged saccadic latency in YOD, compared with controls and FCD, provides objective evidence of oculomotor dysfunction associated with neurodegenerative changes affecting cortical networks involved in attentional control and disengagement.^23^ The graded increase in latency from Controls to FCD to YOD suggests a continuum of functional decline, with some individuals with FCD potentially representing a prodromal stage, while others remain below the diagnostic threshold for YOD.^6^^,^^24^^,^^25^

Functional cognitive disorder is increasingly recognized as a spectrum disorder, where most patients do not progress to dementia, yet a subset-particularly those with depression, post-traumatic stress disorder, or delirium, may be at higher risk.^6^^,^^26^^,^^27^ Shared neurobiological features, including altered brain-derived neurotrophic factor levels^28^, ^29^, ^30^ and reduced acetylcholine, may contribute to vulnerability to cognitive decline.^31^ Prior biomarker studies have largely compared dementia with healthy controls; FCD, its key clinical differential, has rarely been used as a comparator, a recent exception being saccadic measures.^8^ Our multimodal, point-of-care approach extends this, with FCD consistently intermediate between controls and YOD. This intermediate position, together with the heterogeneity of our FCD cohort, including individuals with depression and chronic conditions, likely underlies the lack of significant differences between groups, with some patients resembling controls and others early YOD. These findings highlight the need for longitudinal studies to determine whether neuro-ophthalmic markers can predict progression from FCD to dementia and support risk stratification and personalized monitoring. The heterogeneity we observed highlights the importance of viewing FCD not as a simple exclusion diagnosis but as a complex condition requiring nuanced clinical assessment and, potentially, biomarker-assisted prognostication.

Both vertical (9°) and horizontal (20°) paradigms demonstrated delayed initiation in YOD, reflecting reduced efficiency within neural circuits responsible for saccadic generation. Saccadic eye movements are governed by a distributed cortical–subcortical network involving the primary and extrastriate visual cortices, posterior parietal regions, frontal eye fields, and supplementary eye fields within the frontal cortex, alongside subcortical structures such as the striatum, thalamus, and superior colliculus,^32^ which are disrupted in AD,^23^^,^^33^ resulting in delayed saccades and increased errors.^13^^,^^34^ These saccadic changes may also reflect attentional deficits, supported by associations between saccadic parameters and cognitive performance (as measured with the Mini Mental State Exam), underscoring their potential clinical value.^23^ Oculomotor measures therefore hold promise as objective biomarkers for differentiating early dementia states.^23^^,^^35^^,^^36^ Our finding of prolonged saccadic latency in YOD is consistent with meta-analytic evidence in neurodegenerative cognitive impairment,^35^ while the intermediate position of our FCD group is in keeping with recent evidence that saccadic measures can distinguish FCD from cognitive impairment.^8^

Although some studies suggest vertical saccades are more vulnerable to neurodegeneration,^37^^,^^38^ our data demonstrated more distinct latency prolongation in the horizontal paradigm, consistent with prior work and theoretically linked to dysfunction in posterior cortical regions.^36^ Horizontal saccades may thus be more sensitive to early impairments, capturing subtle predementia dysfunction. Collectively, these findings suggest that saccadic latency represents a biomarker capable of distinguishing YOD from nondementia participants and identifying prodromal functional decline.

Pupillometric outcomes highlight the potential of dynamic pupil responses as sensitive markers of neurodegenerative change. Although baseline pupil diameter and RAPD did not differ significantly between groups, both measures demonstrated a stepwise decline from Controls and FCD to YOD. Pupil diameter is primarily regulated by the autonomic nervous system and influenced by neuromodulatory centers such as the locus coeruleus and Edinger–Westphal nucleus,^39^ as well as by light detected by classical photoreceptors and melanopsin-expressing intrinsically photosensitive retinal ganglion cells.^40^ Given that AD-related neurodegeneration affects both autonomic contributions to the pupillary light reflex and the afferent limb via intrinsically photosensitive retinal ganglion cells dysfunction,^14^ the directionally consistent reductions in baseline diameter and RAPD observed in our cohort are physiologically plausible. The mixed findings in the literature further contextualize our results: some studies report limited diagnostic sensitivity of baseline pupil diameter,^41^ and even of constriction dynamics,^42^ whereas others demonstrate significant differences between neurodegenerative and healthy groups.^14^ In this context, the downward trend in our cohort may reflect early parasympathetic or sympathetic dysfunction, intracellular retinal changes, or early intrinsically photosensitive retinal ganglion cells involvement affecting afferent pathway.

Dynamic pupillary metrics, in contrast, showed robust group effects. Young-onset dementia participants had markedly slower constriction velocity and acceleration compared with controls + FCD. Consistent with the literature, pupillometric slowing reflects cholinergic dysfunction, particularly within the Edinger–Westphal nucleus, in early neurodegenerative disease, which may explain the distinct deterioration observed in constriction parameters; indeed, maximum constriction velocity and acceleration are among the most sensitive indicators of cholinergic impairment.^41^^,^^43^ These reductions are consistent with prior pupillometric findings and extend recent handheld quantitative pupillometry^44^ to a rapid, point-of-care, young-onset setting. When analyzed across all 3 cognitive groups, a stepwise decline in constriction dynamics was evident. As discussed previously, this may be related to the heterogeneity within the FCD cohort. However, it is important to note that the FCD group also carried the highest anticholinergic burden, and muscarinic blockade directly attenuates pupillary constriction, so a medication contribution to these intermediate values cannot be excluded. We therefore present the pupillometric gradient as an observation requiring confirmation rather than as evidence of a cognitive continuum. Overall, our findings support dynamic pupillometry, particularly constriction velocity and acceleration, as biomarkers capable of distinguishing YOD from nondementia groups, with the potential to capture subtle functional deterioration across stages of cognitive impairment and a tool to monitor progression.^14^^,^^41^^,^^43^

Our ERG-PhNR analysis was limited by insufficient statistical power, a recognized constraint of this feasibility study design. Although amplitude and latency differences did not reach statistical significance between groups, the trend toward reduced amplitude and prolonged latency in the YOD group compared with nondementia group suggests subtle functional alterations within the inner retinal pathways. Post hoc power analysis indicated that approximately 45 participants per group would be required to achieve 80% power at α = 0.05 for the observed effect size (Cohen's d ∼ 0.35 for latency). Importantly, detecting the small amplitude differences observed (d ∼ 0.25) would require prohibitively large samples (>100 per group), suggesting that PhNR amplitude may be less sensitive than latency as an early biomarker, or that the signal is genuinely subtle at this disease stage. These considerations should inform the design of future validation studies, which may benefit from larger multicenter cohorts, standardized recording protocols, and longitudinal follow-up to establish whether PhNR changes predict cognitive decline.

Minor latency delays may reflect early dysfunction of inner retina-retinal ganglion cells or their downstream connections,^45^^,^^46^ consistent with the sensitivity of PhNR to neurodegenerative processes in AD.^16^ The trend toward prolonged latency in our cohort differs from the predominantly reduced PhNR amplitude reported in moderate AD.^47^ This divergence may reflect differing sensitivity of PhNR parameters across disease stages, with latency changes emerging earlier in our younger, less severely affected cohort and amplitude reductions becoming prominent later; both, however, point to inner retina involvement. Across groups, latency increased progressively from controls to YOD, with FCD falling in between, which likely reflects FCD heterogeneity.

Dementia is a global health challenge, and early, equitable diagnosis remains essential yet difficult, particularly given the linguistic, cultural, and educational biases of standard cognitive tests.^48^ Accurate differentiation between FCD and dementia is especially important in younger adults to ensure appropriate and timely referral pathways.^49^ Within this context, our findings demonstrate that language-independent visual pathway assessments are both well-tolerated and capable of detecting functional decline in oculomotor and pupillary responses, with more variable but encouraging signals from retinal electrophysiology. Participant feedback further confirmed high acceptability, with most individuals rating the procedures as comfortable and appropriately paced, supporting the feasibility of integrating these tools into routine clinical and research workflows, particularly in memory clinics, where fatigue, reduced attention, and sensory sensitivity are common.^20^^,^^50^ Overall, these results highlight the promise of neuro-ophthalmic biomarkers as objective, background-fair measures that could strengthen diagnostic pathways, enhance equity in dementia care, and facilitate earlier identification of individuals at risk.

A key strength of this study is the integration of noninvasive, point-of-care neuro-ophthalmic tools within a routine clinical setting. The multimodal approach enabled concurrent assessment of cortical, autonomic, and retinal function, providing a more comprehensive understanding of visual pathway changes in cognitive decline. High levels of patient acceptability further highlight the feasibility of implementing these tests in routine practice, which is particularly valuable in memory clinics where time constraints and patient tolerability are critical considerations.

Nevertheless, several limitations must be acknowledged. First, the modest sample size limited statistical power, particularly for subgroup comparisons, and contributed to the nonsignificant ERG-PhNR findings. Second, both patient groups were clinically heterogeneous: the YOD group spanned 6 diagnoses with differing oculomotor and pupillary involvement and was too small to stratify, so our mechanistic interpretations reflect group-level tendencies that may not apply uniformly across subtypes; and the FCD group, although classified on the basis of internal inconsistency, included participants with comorbidities that may have contributed to their cognitive symptoms, so findings in this group should be interpreted with caution. Third, controls were a convenience sample recruited from relatives and staff, predominantly of White ethnicity and without recorded education data, so validation in more diverse cohorts is needed before equity claims can be substantiated. Fourth, only cross-sectional data were collected, without concurrent cerebrospinal fluid or blood-based assays, precluding assessment of longitudinal change and additive diagnostic utility. Finally, anticholinergic burden was highest in the FCD group, with 6 participants scoring ≥3 in total (5 with FCD and 1 with YOD). Therefore, potential confounding effects of sex, medication use, including cholinesterase inhibitors, and systemic comorbidities cannot be fully excluded. Given the small subgroup sizes, the present study was not designed or powered to undertake detailed medication-stratified or medication-controlled analyses, highlighting the need for larger prospective studies incorporating detailed medication data and medication-controlled analyses. The acceptability questionnaire was completed by only 67% of participants, with lower completion among patients; although largely attributable to clinic time constraints, responder bias cannot be excluded. As the study focused on YOD, participants were relatively young, and generalizability to older populations requires further study.

In conclusion, this study demonstrates that multimodal, noninvasive neuro-ophthalmic assessments are feasible, well tolerated, and capable of objectively capturing early functional decline as culture-fair tools. Pupillometry and saccadic latency emerged as promising and differential biomarkers of early impairment with improved diagnostic performance when combined, supporting the value of a multimodal approach. Electroretinography photopic negative response findings were inconclusive but suggest potential retinal involvement requiring further study. Future research should validate these findings and assess their predictive value for early diagnosis and monitoring of dementia.
