# Supplementary material for: Feasibility and Diagnostic Potential of Multimodal Neuro-Ophthalmic Biomarkers in Young-Onset Dementia and Functional Cognitive Disorder
Source: Ophthalmol Sci. 2026 Sep 17;6(11):101410. doi: 10.1016/j.xops.2026.101410 (PMC13693086; doi:10.1016/j.xops.2026.101410)
Supplement: Supplementary file 1 [file mmc1.pdf]

Supplementary Data 1: Participant Experience

A total of 30 participants (Controls = 15, FCD = 6, YOD = 9) evaluated their examination experience using a 5-point Likert scale, where 5 indicated "strongly agree" and 1 indicated "strongly disagree."

Participant Experience Assessment Questionnaire

A. I felt comfortable during the eye tests and the head positioning required for the examination was easy to maintain.

B. The length of the eye examination was appropriate.

C. The brightness levels used during the examination were comfortable for my eyes.

D. I did not experience double vision at any point during the examination or any of the tests conducted.

E. I did not experience eye strain or fatigue during or at the end of the examination.

The questionnaire was completed by 30 of 45 participants (67%): 15/18 controls (83%), 6/11 FCD (55%) and 9/16 YOD (56%). Completion did not differ significantly across groups ($\chi^2(2) = 3.76$, $p = 0.15$). Among completers, the majority reported favourable experiences: 96.7% found the test comfortable, 96.7% considered the examination length appropriate, 93.3% were satisfied with brightness, 90.0% reported no eye strain or fatigue, and 63.3% reported no double vision (Supplementary Figure S1). Acceptability scores did not differ significantly between groups on any item after Bonferroni correction (Supplementary Table S1); the only nominal difference (comfort, $H = 7.50$, $p = 0.024$) did not survive correction and was of negligible magnitude (all group means ≥ 4.56 of 5).

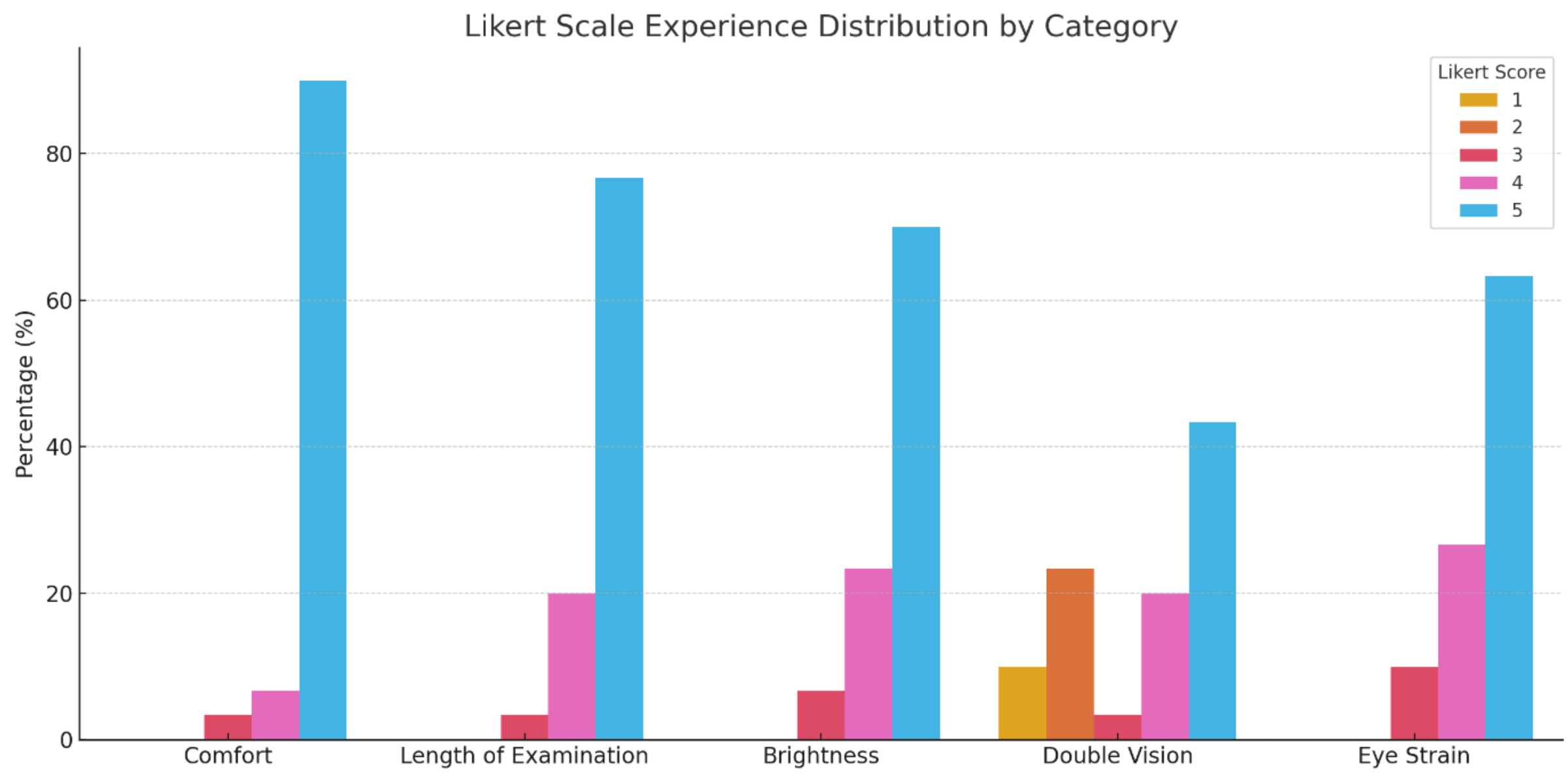

Supplementary Figure S1: Patient Experience Across Different Variables. *Bar charts illustrating the proportion of participants rating across five different aspects of the examination experience: comfort, appropriateness of examination length, satisfaction with brightness levels, absence of double vision and absence of eye strain/fatigue.*

Question	Controls (n = 15)	FCD (n = 6)	YOD (n = 9)	H	p
Comfort	5.00	5.00	4.56	7.50	0.024
Examination length	4.67	4.83	4.78	0.31	0.86
Brightness	4.60	4.67	4.78	1.12	0.57
Absence of double vision	3.53	3.67	3.67	0.27	0.88
Absence of eye strain / fatigue	4.53	4.50	4.56	0.21	0.90

Supplementary Table S1. Acceptability by group among questionnaire completers. *Values are mean Likert scores (1–5; higher = more favourable). H and p from the Kruskal–Wallis test; after Bonferroni correction for five comparisons.*
