# Supplementary material for: Feasibility and Diagnostic Potential of Multimodal Neuro-Ophthalmic Biomarkers in Young-Onset Dementia and Functional Cognitive Disorder
Source: Ophthalmol Sci. 2026 Sep 17;6(11):101410. doi: 10.1016/j.xops.2026.101410 (PMC13693086; doi:10.1016/j.xops.2026.101410)
Supplement: Supplementary file 2 [file mmc2.pdf]

Supplementary Data: Eye-tracking calibration and data acquisition

Saccadic and pupillometric measurements were obtained using the BulbiCAM system. Saccadic measurements were calibrated using an internal two-point procedure embedded within the task. For each saccade, calibration relied on assumed stable fixation at two time points: initial fixation on the central target before the saccade, and fixation on the peripheral target after completion and stabilisation of the eye movement. The known angular separation between these targets was mapped to the corresponding glint-based eye-tracking signal at these two moments, and all gaze samples recorded between them were linearly adjusted to convert pixel displacement to degrees of eye rotation. This segment-wise recalibration was repeated for each saccade, allowing compensation for gradual changes in pupil–camera geometry during the trial. Blink-contaminated samples were excluded prior to calibration, and the resulting calibrated gaze signal was used for subsequent analyses of saccade latency, velocity, and accuracy.

Saccadic latency was recorded to evaluate potential functional decline, with vertical 9° and horizontal 20° saccades assessed. According to the device’s standardised test protocol, saccade test consisted of five trials of the vertical saccade task from +9° upward to −9° downward and five trials of the horizontal saccade task from −20° leftward to +20° rightward movements. Figure 1C shows examples of horizontal and vertical saccades. For pupillometry, baseline pupil diameter, relative afferent pupillary defect (RAPD), mean constriction velocity, and mean constriction acceleration were analysed. The diagram in Figure 1D shows the original test outputs. Peak constriction velocity was defined as the maximum negative slope of the pupil diameter trace; absolute values are reported.
