# Supplementary material for: Feasibility and Diagnostic Potential of Multimodal Neuro-Ophthalmic Biomarkers in Young-Onset Dementia and Functional Cognitive Disorder
Source: Ophthalmol Sci. 2026 Sep 17;6(11):101410. doi: 10.1016/j.xops.2026.101410 (PMC13693086; doi:10.1016/j.xops.2026.101410)
Supplement: Supplementary file 3 [file mmc3.pdf]

Supplementary Data 3: Diagnostic Performance of Oculomotor and Pupillometric Markers

Receiver Operating Characteristic (ROC) analyses were conducted to evaluate the ability of oculomotor and pupillometric parameters to discriminate between young-onset dementia (YOD) and non-dementia participants (Controls+FCD).

Overall, several parameters demonstrated moderate diagnostic performance. Pupillary constriction acceleration showed the highest discriminative ability (AUC = 0.83), followed by horizontal saccadic latency (AUC = 0.77). Pupillary constriction velocity (AUC = 0.72) and vertical saccadic latency (AUC = 0.70) demonstrated comparatively lower specificity despite relatively high sensitivity.

Optimal cut-off values were determined using the Youden Index to balance sensitivity and specificity. At these thresholds, horizontal saccadic latency showed the highest sensitivity (85.7%), while pupillary constriction acceleration provided the most balanced diagnostic performance (sensitivity 76.9%, specificity 74.1%).

Detailed diagnostic metrics, including sensitivity, specificity, and optimal cut-off values, are presented in Supplementary Table S3. Corresponding ROC curves are shown in Supplementary Figure S3.

Supplementary Table S3: Diagnostic performance of oculomotor and pupillometric parameters

Parameter	AUC (95% CI)	Sensitivity (%)	Specificity (%)	Cut-off
Horizontal Saccadic Latency (20°)	0.77 (0.59–0.95)	85.7	75.9	>304.5 ms
Vertical Saccadic Latency (9°)	0.70 (0.53–0.86)	80	58.6	>218.5 ms
Pupillary Constriction Acceleration	0.83 (0.69–0.96)	76.9	74.1	< 2.2
Pupillary Constriction Velocity	0.72 (0.55–0.89)	84.6	55.6	< 1.6

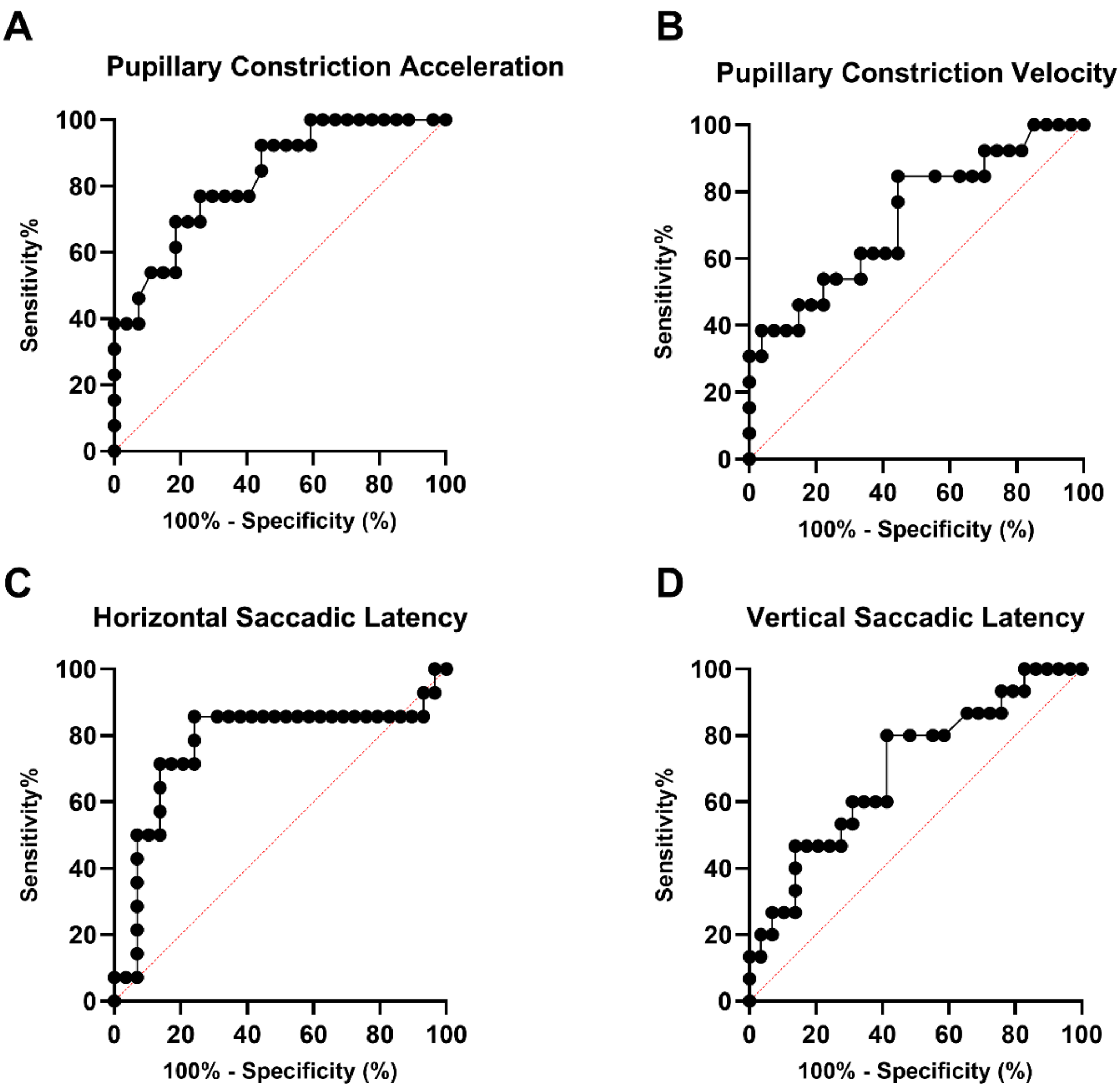

Supplementary Figure S3: ROC curves of oculomotor and pupillometric parameters for discriminating YOD from non-dementia participants. (A) Pupillary constriction acceleration, (B) pupillary constriction velocity, (C) horizontal saccadic latency (20°), and (D) vertical saccadic latency (9°). The dashed line represents chance level.
