# Supplementary material for: Feasibility and Diagnostic Potential of Multimodal Neuro-Ophthalmic Biomarkers in Young-Onset Dementia and Functional Cognitive Disorder
Source: Ophthalmol Sci. 2026 Sep 17;6(11):101410. doi: 10.1016/j.xops.2026.101410 (PMC13693086; doi:10.1016/j.xops.2026.101410)
Supplement: Supplementary file 4 [file mmc4.pdf]

Supplementary Data 4: Combined Biomarker Model

A combined biomarker model including pupillary constriction acceleration and horizontal saccadic latency demonstrated good discriminatory performance (AUC = 0.84, 95% CI: 0.70–0.98).

Classification analysis showed an overall accuracy of 82.5%, with high specificity (92.6%) and moderate sensitivity (61.5%). The positive predictive value was 80.0%, and the negative predictive value was 83.3%.

These findings suggest that combining biomarkers reflecting distinct physiological domains may provide complementary diagnostic information. However, given the sample size, these results should be interpreted as exploratory and require validation in larger cohorts.

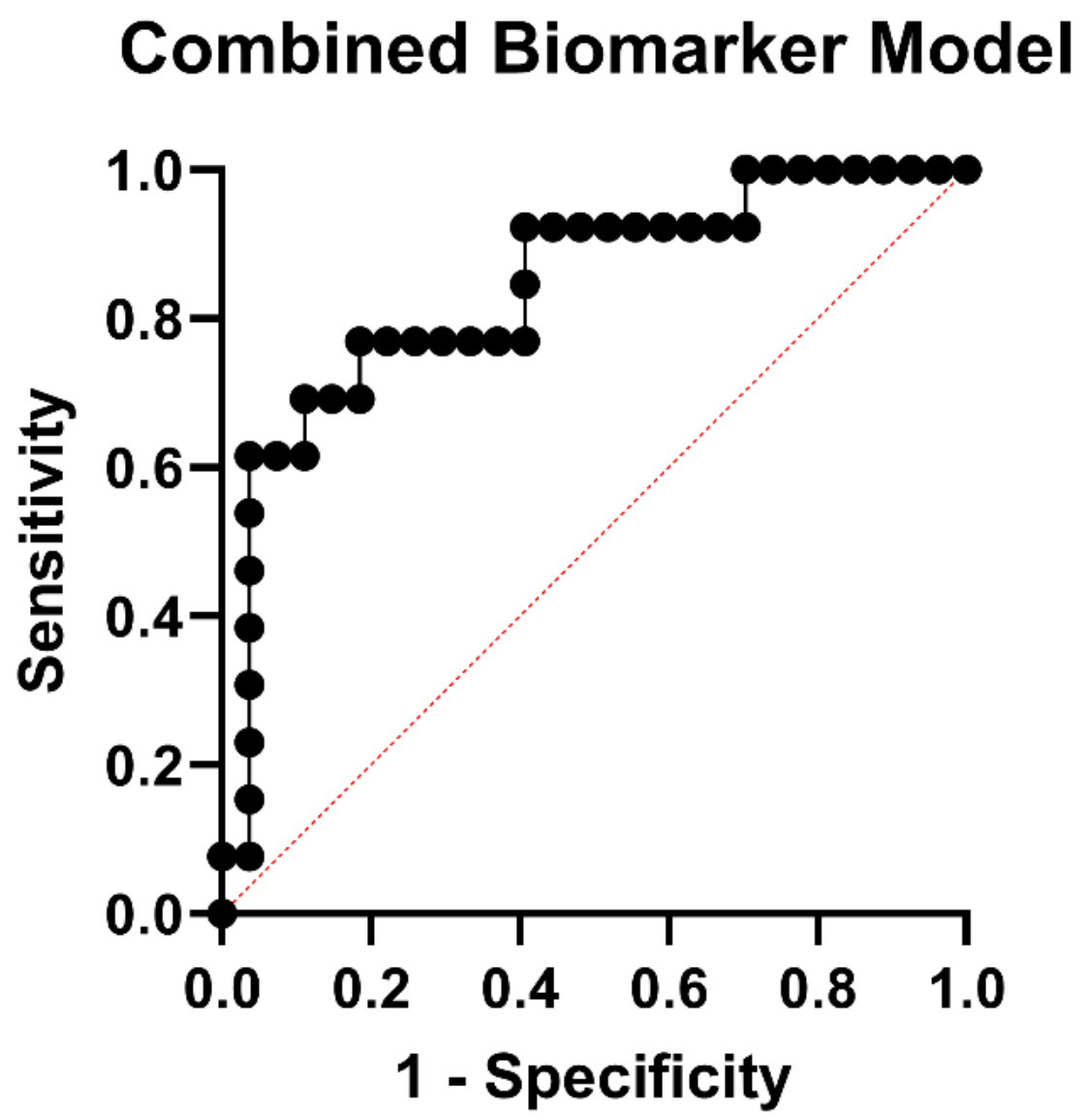

Supplementary Figure S4: Combined ROC curve. ROC curve of the combined biomarker model including pupillary constriction acceleration and horizontal saccadic latency. The model demonstrates improved discrimination between young-onset dementia and non-dementia participants. The dashed line represents chance level (AUC = 0.5).

Supplementary Table S4: Classification performance of the combined model

**AUC**	0.84 (95% CI: 0.70–0.98)
**Accuracy**	82.50%
**Sensitivity**	61.50%
**Specificity**	92.60%
**Positive Predictive Value (PPV)**	80.00%
**Negative Predictive Value (NPV)**	83.30%
